# The Role of Proenkephalin in Predicting Renal Dysfunction and Mortality in Septic Patients Admitted to the Emergency Department and Intensive Care Unit: A Narrative Review

**DOI:** 10.3390/jpm16090451

**Published:** 2026-08-28

**Authors:** Christos Verras, Ioannis Ventoulis, Sofia Bezati, John Parissis, Effie Polyzogopoulou

**Affiliations:** 1 Department of Emergency Medicine, Attikon University Hospital, National and Kapodistrian University of Athens, Rimini 1, 12462 Athens, Greece; sofiabezati@gmail.com (S.B.); jparissis@yahoo.com (J.P.); effiepol@med.uoa.gr (E.P.); 2 Department of Occupational Therapy, University of Western Macedonia, Keptse Area, 50200 Ptolemaida, Greece; iventoulis@uowm.gr

**Keywords:** proenkephalin, PENK, biomarker, sepsis, septic shock, emergency department, intensive care unit

## Abstract

Prompt diagnosis and treatment of sepsis and septic shock is crucial due to the persistently high rates of morbidity and mortality associated with the disease. Recently, research efforts have focused on identifying novel biomarkers that would detect, classify, and assess the severity of sepsis in a timely manner, thereby allowing for expeditious and precise treatment of sepsis and septic shock. Among these biomarkers, proenkephalin (PENK) has gained considerable attention. Accordingly, this narrative review aims to consolidate current evidence on the clinical utility of PENK in patients with sepsis and septic shock, with emphasis on its role as a biomarker of renal dysfunction and as a predictor of sepsis-associated acute kidney injury (AKI) and mortality in both the emergency department (ED) and intensive care unit (ICU) settings.

## 1. Introduction

Despite advances in diagnosis and management, sepsis continues to impose a substantial global healthcare burden owing to its persistently high morbidity and mortality [1,2,3,4]. Septic shock is the most advanced and life-threatening manifestation of sepsis, characterized by refractory hypotension requiring vasopressor support despite adequate fluid resuscitation, and accompanied by impaired tissue perfusion and elevated serum lactate levels. The resulting circulatory and metabolic derangements are indicative of severe organ dysfunction and are closely linked to the markedly elevated mortality risk in this patient population [5,6,7,8].

Moreover, the clinical burden of sepsis extends considerably beyond the initial acute episode, as many survivors often face substantial long-term morbidity with persistent and debilitating symptoms. Indeed, some patients progress to a state of chronic critical illness, marked by sustained low-grade inflammation and immunosuppression, accelerated protein catabolism, progressive loss of lean tissue, and cachexia, ultimately leading to prolonged functional limitations and impaired quality of life [9,10,11,12,13]. It has been estimated that approximately one third of sepsis survivors die within a year, while 40% are re-hospitalized within 3 months of their initial discharge [14].

In response to the substantial global burden of sepsis, numerous international initiatives, including the World Health Organization (WHO) resolution adopted at the 70th World Health Assembly [15], have been undertaken to improve prevention strategies, facilitate early diagnosis and optimize timely and effective treatment [16,17]. Despite these advances, the clinical management of sepsis remains largely standardized, underscoring the urgent need to move towards individualized and patient-centered approaches that can improve diagnostic accuracy and therapeutic decision-making [18,19,20]. In recent years, growing efforts have focused on identifying reliable biomarkers that can capture the complex host response to sepsis and enable early diagnosis, timely detection of organ dysfunction, accurate risk stratification, and precise prognostication [21,22,23,24,25,26].

The aim of this narrative review is to critically assess the role of proenkephalin (PENK) as a biomarker in septic patients presenting to the Emergency Department (ED) or admitted to the Intensive Care Unit (ICU), with particular emphasis on its association with renal dysfunction and mortality. This review first provides an overview of the molecular biology and pathophysiological basis of PENK, followed by a comprehensive synthesis of clinical studies investigating its clinical utility in sepsis across ED and ICU settings. Particular attention is placed on its potential applications as a tool for early identification of renal dysfunction and sepsis-associated acute kidney injury (AKI), risk stratification, and prediction of mortality and other clinically relevant adverse outcomes.

To this end, a literature search was conducted to identify relevant peer-reviewed publications addressing the role of PENK in sepsis and septic shock. PubMed (MEDLINE) and Scopus were searched from database inception through 15 February 2026, without restrictions on publication date. The search strategy included combinations of the following terms: “proenkephalin”, “PENK”, “sepsis”, “septic shock”, and “biomarkers”, using Boolean operators as appropriate. The search was restricted to articles published in English and involving human subjects. Retrieved publications were screened based on their titles and abstracts to identify studies relevant to the objectives of this review. Articles considered potentially relevant were subsequently evaluated in full text. Studies were considered eligible when they investigated PENK in patients with sepsis or septic shock and provided information relevant to its diagnostic, prognostic, renal, or clinical significance. Studies focusing exclusively on non-septic populations, as well as in vitro, experimental, and animal studies, were excluded. Additional relevant studies were identified through manual screening of the reference lists of selected articles and relevant review papers. Given the narrative nature of this review, study selection was based on relevance of the available evidence to the predefined scope and objectives of the review rather than a formal systematic-review protocol.

## 2. Molecular Biology and Pathophysiology of PENK

Enkephalins are endogenous opioids that exert their biological effects through binding to μ (mu)-, κ (kappa)-, and δ (delta)- opioid receptors. Leucine-enkephalin (Leu-enkephalin) and Methionine-enkephalin (Met-enkephalin) are the two major active forms of enkephalins, which are derived from a 267-amino-acid-long precursor protein, termed preproenkephalin A (pPENK-A). Active enkephalins have a very short in vitro half-life (approximately 15 min) [27,28,29,30]. Although the exact physiological functions of enkephalins remain incompletely understood, studies in both humans and animal models have demonstrated their involvement in cardiovascular regulation, particularly in cardiac contractility and hemodynamic control, as well as in renal function [27,28,30,31,32]. Specifically, activation of opioid receptors by enkephalins exerts cardiodepressive effects on the myocardium by reducing myocardial contractility, blood pressure, and heart rate. Furthermore, enkephalins inhibit sympathetic activity by suppressing norepinephrine release and attenuating sympathetic vasoconstriction. Enkephalins have also been implicated in mediating cardioprotection through ischemic preconditioning [28,30,32,33]. Additionally, they appear to play a regulatory role in kidney function, by increasing renal blood flow and urinary output, and by promoting diuresis and natriuresis either through central inhibition of the antidiuretic hormone (ADH) secretion or through peripheral inhibition of ADH action [27,28,30,31].

PENK is a biologically inactive byproduct generated during the proteolytic cleavage of the precursor molecule pPENK-A. It is a relatively large monomeric peptide comprising 41 amino acids (residues 119–159) with a small molecular mass of 4.5 kDa. Unlike the biologically active enkephalins, which are unstable and difficult to quantify in plasma due to their short half-life, PENK possesses a considerably longer half-life of approximately 48 h. This property confers greater in vitro stability and enables reliable and reproducible measurements in plasma. Accordingly, PENK has emerged as a stable surrogate biomarker of the endogenous enkephalin activity [27,28,29,30].

PENK has been shown to be closely associated with kidney function, as it appears to be freely filtered through the glomerulus. Elevated circulating PENK levels have been consistently associated with worsening renal function and the development of AKI. More importantly, PENK seems to reflect real-time glomerular filtration rate (GFR), even under non-steady-state conditions, remaining largely unaffected by critical illness, systemic inflammation, age or sex. These characteristics render PENK a promising functional biomarker of renal function [27,31].

Accumulating evidence further suggests that PENK is an early predictor of AKI and may also be useful for predicting AKI duration and renal recovery [27,31]. Consistent with these findings, the 23rd Acute Disease Quality Initiative (ADQI) consensus conference recommended the use of novel biomarkers, including PENK, as adjuncts to clinical assessment for predicting AKI progression and recovery. However, this recommendation was graded as weak, reflecting the need for further validation in prospective clinical studies [34].

Beyond its role as a marker of renal dysfunction, elevated PENK levels have also been associated with adverse renal and cardiovascular outcomes, including major adverse cardiovascular events, renal replacement therapy (RRT), hospital readmission and all-cause mortality. These associations have been reported across a wide range of clinical settings, including sepsis, heart failure, chronic kidney disease, cardiac surgery, and myocardial infarction [27,30,31,32,35,36,37,38,39,40,41]. Within this spectrum of clinical conditions, sepsis represents a particularly relevant setting, given the high incidence of renal dysfunction and its well-established association with adverse outcomes. This has generated interest in PENK as a potential biomarker for early risk stratification and prognostic assessment in septic patients.

## 3. PENK as a Marker of Renal Dysfunction in Sepsis

Given the close relationship between PENK and renal function, most studies investigating its role in sepsis have focused on its ability to identify patients at risk of kidney injury and adverse renal outcomes. Accordingly, all reviewed studies [41,42,43,44,45,46,47,48,49,50,51,52,53,54] evaluated PENK as a biomarker for risk stratification and disease severity, with particular emphasis on its association with renal dysfunction. Specifically, these studies assessed PENK’s ability to predict AKI, major adverse kidney events, and the need for RRT in patients admitted to the ED and/or the ICU.

AKI is a frequent and life-threatening complication of sepsis that imposes a substantial burden on healthcare systems and is associated with considerable morbidity and mortality. Compared to AKI of other etiologies, sepsis-associated AKI is typically more complex, rapidly evolving, and severe. Sepsis-associated AKI is multifactorial in nature and arises from a complex interplay of pathophysiological mechanisms. Microcirculatory impairment, glomerular ischemia–reperfusion injury, endothelial dysfunction, coagulopathy, and systemic and renal inflammation play important roles in initiating renal damage. This process is further driven by cellular metabolic reprogramming, hypoxia, oxidative stress, cytokine- and chemokine-mediated tubular injury, and cellular apoptosis. Hemodynamic instability and renal hypoperfusion, particularly in septic shock, may further aggravate kidney damage. The convergence of these mechanisms ultimately contributes to the high incidence and severity of AKI observed in patients with sepsis [55,56,57,58,59]. This close pathophysiological link between sepsis and AKI provides the biological rationale for evaluating PENK as an early biomarker of renal dysfunction and adverse renal outcomes in septic patients.

A prospective observational study [42] enrolled 42 postoperative ICU patients with sepsis to evaluate the ability of PENK to predict and monitor kidney function. Patients were classified into three subgroups according to sepsis severity (sepsis, severe sepsis, septic shock). PENK levels, measured within 16 h after the diagnosis of sepsis, showed a moderate positive correlation with serum creatinine and a strong negative correlation with estimated GFR (eGFR). In a multivariable linear regression analysis including age, sex, body mass index (BMI), eGFR, renal dysfunction and metabolic acidosis, eGFR emerged as the strongest independent determinant of plasma PENK concentrations. PENK levels did not differ significantly among the subgroups; however, patients with renal dysfunction and metabolic acidosis had significantly higher PENK concentrations. Taken together, these findings suggest that PENK may represent a useful biomarker for monitoring kidney function in surgical ICU patients with sepsis [42].

An interesting endpoint was set by a pilot study [43], which aimed to examine the relationship between plasma PENK concentrations and the “true” GFR determined by iohexol plasma clearance (GFR_iohexol_) under non-steady-state conditions. The study also evaluated whether PENK could estimate GFR more accurately than conventional creatinine-based methods, namely eGFR derived from the Modification of diet in renal disease study (MDRD) equation (eGFR_MDRD_) and GFR measured by endogenous creatinine clearance (GFR_ECC_). For this purpose, they recruited 23 critically ill patients admitted to the ICU with sepsis, of whom 20 were in septic shock. Although both eGFR_MDRD_ and GFR_ECC_ were correlated with the “gold standard” GFR_iohexol_, both methods tended to overestimate renal function, resulting in misclassification of GFR categories. PENK concentrations were inversely correlated with GFR_iohexol_ and demonstrated a stronger association with the reference method (GFR_iohexol_) than both conventional creatinine-based methods. The authors concluded that PENK may serve as an accurate and reliable biomarker of renal function in critically ill septic patients, since it seems to reflect renal function more precisely even under non-steady-state conditions [43].

Expanding on these findings, a retrospective observational study [44] evaluated the performance of PENK and neutrophil gelatinase-associated lipocalin (NGAL), measured upon admission to the ED, in terms of detecting the presence and severity of AKI in 101 patients with suspected sepsis. AKI severity was determined according to the RIFLE criteria (risk, injury, failure, loss of function, end-stage renal disease). Consistent with the previous study, PENK concentrations were strongly associated with renal function, demonstrating a significant inverse correlation with creatinine clearance. Furthermore, multivariable linear regression identified creatinine clearance as the strongest independent predictor of circulating PENK levels, whereas cardiovascular disease and mean arterial pressure exerted only modest effects. PENK concentrations were significantly higher in patients with severe sepsis or septic shock than in those with sepsis and increased progressively with AKI severity. More importantly, median PENK concentrations remained low in patients without AKI and in those at risk for AKI, whereas they increased markedly in patients with kidney injury, renal failure, and loss of kidney function. NGAL demonstrated a similar increase across the RIFLE stages and comparable diagnostic accuracy for AKI, with an area under the curve (AUC) of 0.800 compared to 0.815 for PENK. However, unlike PENK, NGAL concentrations appeared to be substantially influenced by the systemic inflammatory response associated with sepsis, since nearly all patients without AKI exhibited NGAL concentrations above the normal cut-off (0.1 μg/mL), whereas the corresponding PENK concentrations remained within the normal range (below 80 pmol/L). Furthermore, although both biomarkers demonstrated similar diagnostic performance for AKI, PENK provided significant incremental predictive value beyond that offered by NGAL alone. Notably, the predictive information provided by PENK was independent of NGAL, suggesting that the two biomarkers may capture distinct and potentially complementary aspects of renal dysfunction. The combination of PENK and NGAL therefore improved the identification of kidney dysfunction, with the addition of PENK to NGAL resulting in a significant improvement in predictive performance (incremental χ^2^ = 10.0; *p* = 0.0016). Collectively, these findings indicate that PENK may be a more specific biomarker than NGAL for assessing AKI in septic patients, given that its circulating concentrations seem to be less affected by systemic inflammation while maintaining comparable diagnostic performance for AKI [44].

These findings were corroborated by a retrospective study conducted by Kim et al. [45] in 167 septic patients, of whom 37 were classified as having septic shock, 99 as sepsis and 31 as suspected sepsis. The aim of the study was to explore the diagnostic and prognostic utility of PENK in comparison to NGAL and eGFR. Renal function was assessed using four different eGFR equations: the MDRD equation, and three Chronic kidney disease epidemiology collaboration (CKD-EPI) equations based on serum creatinine, serum cystatin C or the combination of both biomarkers. PENK, NGAL, and all four eGFR values were found to be significantly correlated with sepsis severity. Nevertheless, their ability to discriminate patients with and without AKI differed according to disease severity. At the stage of sepsis, the values of PENK, NGAL and all four eGFR equations were significantly different between patients with and without AKI. On the contrary, at the stage of septic shock, PENK and NGAL levels were elevated and eGFR values were reduced even in patients without AKI, thereby restricting the ability of all biomarkers to discriminate AKI in patients with septic shock. Furthermore, with regard to the stage of sepsis, it was found that PENK levels were not increased in patients who did not develop AKI, whereas NGAL levels were elevated even in patients without AKI, suggesting that PENK levels do not seem to be influenced by the inflammatory process as opposed to NGAL [45]; an observation which is consistent with the findings of Marino et al. [44]. When examining the diagnostic performance for AKI using receiver operating characteristic (ROC) curve analysis, PENK and the four eGFR equations exhibited better discriminatory ability for AKI (with AUCs ranging from 0.712 to 0.761) than NGAL (AUC 0.675), although these differences did not reach statistical significance [45]. However, when applying nesting logistic regression models, PENK was found to be superior to NGAL in terms of predicting AKI, but inferior to all eGFR equations except for the CKD-EPI equation based on serum cystatin C. It was also noted that PENK and NGAL levels were significantly higher in patients requiring RRT than in those who did not, with PENK demonstrating better predictive performance for RRT than NGAL (AUC 0.872 versus 0.741, respectively; *p* = 0.0085). Another important finding of the study was that, regardless of the eGFR equation used, PENK demonstrated consistent and significant associations with all four eGFR equations, making it a reliable and objective surrogate marker of reduced GFR and AKI in patients with sepsis [45].

To further investigate the clinical utility of PENK in assessing sepsis severity and predicting adverse clinical outcomes, including organ failure, the same researchers conducted a prospective study involving 215 septic patients from the ICU and ED settings [46]. This patient cohort was entirely distinct from that of their previous 2017 study [45], with no overlap in patient populations and separate enrollment periods [45,46]. The study results, published three years later, were promising, demonstrating that PENK could predict the occurrence of organ failure and the need for RRT [46]. Specifically, PENK levels on admission were significantly higher in patients with septic shock, as well as in those with hemodynamic instability requiring vasopressor support. Moreover, a dose–response relationship between PENK levels and renal dysfunction was evident, since PENK levels increased in parallel with the deterioration of the sequential organ failure assessment (SOFA) renal subscore and the decline in eGFR. Furthermore, higher baseline PENK levels were significantly associated with the need for RRT on the first day of hospitalization. Remarkably, PENK levels were shown to outperform the SOFA renal subscore in predicting the need for RRT. When patients were stratified according to PENK level quartiles, it was observed that the higher the PENK quartile, the worse the renal function and the disease severity, as evidenced by lower eGFR, higher SOFA renal subscore, higher need for RRT, and greater number of organ failures [46].

In addition, the clinical utility of baseline and serial PENK measurements in sepsis has been investigated in a large prospective cohort of 956 ICU patients with sepsis or septic shock [41]. These patients represented a predefined subset of the 1818 participants enrolled in the large multi-center randomized trial called Albumin Italian Outcome Sepsis (ALBIOS) study, which took place across 100 ICUs in Italy. In this subset of patients, plasma PENK concentrations were measured using serial plasma samples collected on days 1 (baseline), 2, and 7 after enrollment, or until ICU discharge if earlier. Patients with septic shock exhibited higher baseline PENK levels than those with sepsis. Moreover, baseline PENK was strongly correlated with serum creatinine and increased progressively along with the deterioration of SOFA renal subscore. Multivariable linear regression analysis demonstrated that baseline PENK was independently associated with markers of renal dysfunction and overall disease severity, including higher serum creatinine levels, lower urinary output, higher total SOFA and Simplified Acute Physiology Score II (SAPS II) scores, chronic kidney disease, mechanical ventilation, lower FiO2 concentration, and lower mean arterial pressure. Furthermore, PENK demonstrated a strong ability to predict the need for RRT. Baseline PENK levels were significantly higher in patients requiring immediate RRT compared to those who either did not require RRT or initiated RRT at a later timepoint during their ICU stay. Apart from that, among patients not receiving RRT at the time of biomarker measurement, PENK not only was an independent predictor of the future need for RRT but also provided incremental prognostic information on top of serum creatinine and other established clinical predictors. What is more, the predictive value of PENK for AKI was once again confirmed. Baseline PENK levels increased progressively with AKI severity and independently predicted the development of both early (within 48 h) and mid-term (within 7 days) AKI, outperforming serum creatinine after multivariable adjustment. Serial measurements further enhanced risk prediction, as PENK measured on day 2 provided additional prognostic value beyond the baseline (day 1) measurement. Patients with persistently low or declining PENK concentrations had a low incidence of AKI, whereas those with persistently elevated or increasing concentrations exhibited a markedly higher risk. Among patients who developed AKI, PENK concentrations remained persistently elevated throughout the first week, regardless of early RRT initiation. Finally, PENK also predicted renal recovery. Lower baseline PENK concentrations were associated with short-term improvement in renal function at both 48 h and 7 days, suggesting that PENK may be useful not only for identifying patients at risk of AKI but also for monitoring AKI progression and recovery [41].

Along the same lines, in the multinational, multicenter Kidney in Sepsis and Septic Shock (Kid-SSS) Study, Hollinger et al. investigated whether PENK could predict major adverse kidney events, AKI, worsening or persistent renal dysfunction, and RRT [47]. Kid-SSS was a predefined substudy of the Adrenomedullin and Outcome in Severe Sepsis and Septic Shock (AdrenOSS) trial and included a cohort of 583 patients who were admitted to the ICU with sepsis or septic shock. The study also used a subgroup of 525 septic patients from the multicenter French and European Outcome Registry in Intensive Care Unit (FROG-ICU) study as a validation cohort. Median [Interquartile range (IQR)] PENK levels upon admission were comparable between the AdrenOSS and the FROG-ICU cohorts, namely 84 (53–156)pmol/L versus 85 (46–156.3) pmol/L, respectively. It was observed that PENK levels increased along with deteriorating SOFA renal subscores, showing a stepwise association with increasing AKI severity. Patients who developed major adverse kidney events (defined as composite of RRT, persistent AKI, and death by day 7) exhibited significantly higher PENK levels at baseline, compared to patients who were free of such events. Likewise, patients with AKI and worsening renal function had significantly higher PENK levels than those without. The validation cohort yielded similar results. PENK levels were also elevated in patients requiring RRT during the first week of hospitalization. Baseline PENK concentrations remained independently associated with major adverse kidney events, AKI and worsening renal function, even after adjustment for potential confounders. Moreover, PENK provided incremental prognostic value beyond serum creatinine, demonstrating greater discriminatory performance for kidney events, particularly among patients with relatively preserved renal function at baseline. Indeed, when analyzing only patients with low baseline creatinine or patients without chronic kidney disease, PENK demonstrated superior predictive performance compared to creatinine. Serial PENK measurements further improved the prediction of major adverse kidney events and worsening renal function. In addition, baseline PENK levels could also predict transient AKI and identify those patients with AKI who were more likely to experience renal recovery [47].

On the contrary, another prospective observational study [48] measured serum PENK concentrations in a cohort of 588 patients presenting to the ED with sepsis and reported that, although baseline PENK levels were independently associated with the development of AKI at 48 h and 7 days, the association between PENK and AKI was attenuated after adjustment for eGFR. Moreover, PENK’s discriminatory performance for AKI was inferior to that of serum creatinine and eGFR. However, when focusing on patients with low renal SOFA scores at presentation, elevated PENK levels could independently predict worsening renal function, even after adjusting for eGFR. Therefore, among patients with normal to mildly impaired renal function upon admission, PENK retained important clinical value by identifying patients with apparently preserved renal function who were at increased risk of subsequent renal deterioration. Moreover, PENK was found to be predictive of multiple organ failure, an association that was maintained after adjustment for eGFR. In fact, PENK demonstrated comparable prognostic performance to creatinine and eGFR for predicting the development of multiple organ failure [48].

Likewise, in a prospective observational study including 40 consecutive septic patients without AKI upon hospital admission, PENK levels were measured at baseline (on admission) and on the 7th day of hospitalization [49]. A total of 16 patients (40%) developed AKI during their hospital stay. PENK levels at baseline showed a weak association with the incidence of AKI, which did not reach statistical significance. However, ROC analysis demonstrated that PENK levels on admission could significantly discriminate septic patients who developed AKI from those who did not (AUC = 0.673; *p* = 0.016), albeit with moderate predictive performance. Apart from that, serial PENK measurements (on day 0 and day 7) demonstrated distinct temporal patterns according to the renal outcome. In patients who did not develop AKI, PENK concentrations decreased significantly over the first week. Conversely, patients who developed AKI exhibited a significant increase in PENK levels over the same period. The latter finding suggests that changes in PENK concentrations over time may provide additional prognostic information on top of baseline measurement, with rising PENK levels potentially reflecting progressive renal dysfunction in septic patients [49].

By the same token, another prospective study comprising 61 patients presenting to the ED with septic shock revealed that PENK levels measured by a point-of-care (POC) device upon ED admission were significantly correlated with metabolic, renal and inflammatory markers [50]. Indeed, PENK showed positive associations with creatinine, procalcitonin, lactate and soluble urokinase plasminogen activator receptor (SuPAR), as well as with SOFA score, implying that PENK could reflect the overall disease severity in septic shock. However, despite these associations, baseline PENK levels did not differ significantly between patients who developed AKI during hospitalization and those who did not, and admission PENK levels were not predictive of AKI occurrence [50].

The prognostic role of PENK in terms of predicting organ dysfunction was evaluated in a multicenter retrospective study [51] which consisted of a large mixed ICU population. Among 1978 patients admitted to four Swedish ICUs, 632 had sepsis, 190 suffered from cardiac arrest, and 157 were trauma patients. Higher PENK levels on admission were associated with greater organ dysfunction on day 2, as evidenced by higher SOFA scores. PENK showed positive associations with renal, cardiovascular, hepatic, and neurological SOFA subscores in the overall ICU population. After adjustment for admission creatinine, PENK remained associated with cardiovascular and neurological dysfunction in the sepsis population, suggesting that its prognostic value extends beyond conventional renal markers. PENK also demonstrated predictive ability for renal dysfunction and RRT requirement on day 2, although creatinine had superior discrimination. Adding PENK to creatinine did not significantly improve prediction of renal failure or RRT use, indicating that PENK may provide complementary information regarding organ dysfunction rather than improving renal risk prediction when creatinine is already available [51].

Recently, a retrospective study [52], including 161 septic patients, was conducted both in the ED and ICU settings. Its aim was to evaluate the ability of PENK to predict sepsis-associated AKI. During hospitalization, 66 patients (41%) developed AKI. Patients who developed AKI exhibited significantly higher PENK levels compared to those who did not. In multivariable analysis, PENK and lactate were identified as independent predictors of AKI. Indeed, elevated levels of lactate and PENK on admission were associated with an increased risk of developing AKI. ROC curve analysis demonstrated that PENK had strong predictive performance for AKI development in ICU patients. The optimal cut-off value for PENK was 2.41 μg/L, with an AUC of 0.867, sensitivity of 78.8%, and specificity of 83.2%. In comparison, lactate showed lower predictive performance, with an AUC of 0.706, sensitivity of 74.2%, and specificity of 61.1% at a cut-off value of 3.45 mmol/L. In other words, PENK demonstrated significantly better predictive ability than lactate. However, adding lactate to PENK did not significantly improve predictive performance compared with PENK alone, indicating that PENK may provide substantial diagnostic value as an independent biomarker for early AKI prediction [52].

The association between PENK and renal dysfunction was further supported by a prospective observational study [53], which included 177 patients presenting to the ED with suspected sepsis. Although the primary endpoint was 30-day mortality, PENK levels on admission showed significant correlations with several markers of disease severity and renal impairment. Higher PENK concentrations were associated with reduced urine output, increased serum creatinine, and higher SOFA scores, as well as lower blood pH and elevated lactate, B-type natriuretic peptide (BNP), and serum potassium levels. These findings suggest that elevated PENK reflects impaired renal function and greater overall physiological derangement in patients with suspected sepsis [53].

To investigate the clinical relevance of PENK in sepsis-associated AKI, Ibrahim et al. conducted a case–control study, which included 50 ICU patients with sepsis-associated AKI and 30 age- and sex-matched healthy controls [54]. The investigators examined the association of PENK with subsequent renal outcomes and mortality. Admission PENK concentrations were significantly higher in patients with sepsis-associated AKI than in controls. ROC curve analysis demonstrated excellent discrimination between the two groups (AUC 0.985), with a sensitivity of 90% and specificity of 100% at the optimal cut-off value of 1200.65 ng/mL. However, the exceptionally high diagnostic accuracy reported for PENK should be interpreted with caution given the case–control design and the lack of a septic non-AKI comparator group. Moreover, PENK levels correlated positively with serum creatinine, C-reactive protein (CRP), systemic inflammatory response syndrome (SIRS) score, and SOFA score. Higher PENK levels were also observed in patients requiring RRT. Furthermore, PENK outperformed serum creatinine, eGFR, and CRP in predicting non-recovery of AKI, defined as chronic kidney disease and/or death. However, it needs to be noted that, unlike the other studies which evaluated PENK in unselected cohorts of septic patients, this study used healthy individuals as the control group. Therefore, these findings should be interpreted within the context of the study design, as the comparison with healthy controls rather than septic patients without AKI may have overestimated the diagnostic performance of PENK [54].

## 4. PENK as a Prognosticator of Mortality

The potential role of PENK in mortality prediction has attracted considerable interest, with studies conducted across ED and ICU settings yielding largely supportive, yet not entirely consistent, results. While elevated PENK concentrations have frequently been associated with increased mortality, its prognostic performance and incremental value over established risk markers have varied considerably among patient populations.

In one of the earliest studies addressing this question, Marino et al. evaluated the prognostic value of PENK and NGAL for short-term mortality in patients presenting to the ED with suspected sepsis [44]. Among the 101 patients included in the study, 28 (28%) died within 7 days of admission. Admission levels of PENK and NGAL were significantly elevated in patients who died early, whereas procalcitonin and creatinine clearance were not predictive of mortality. Despite its strong correlation with creatinine clearance, PENK demonstrated superior prognostic performance for 7-day mortality compared to creatinine clearance (AUC 0.69 vs. 0.61) and, even more so, provided independent prognostic information beyond renal function. NGAL demonstrated comparable prognostic accuracy to PENK (AUC 0.69), whereas the APACHE II score remained the strongest predictor of mortality (AUC 0.75). Notably, serial PENK measurements further improved prognostic discrimination, with the AUC increasing from 0.69 upon admission to 0.90 on day 4 among patients who remained hospitalized. These findings suggest that dynamic changes in PENK concentrations may provide additional prognostic information beyond a single baseline measurement [44].

In their retrospective study, Kim et al. investigated the ability of PENK to predict 30-day mortality and compared its performance to that of NGAL, on one hand, and eGFR, on the other [45]. eGFR was assessed by using 4 different equations: the MDRD formula and three CKD-EPI equations based on serum creatinine, serum cystatin C or both biomarkers. Out of the total 167 septic patients included in the study, 30 subjects (18%) died within 30 days. The authors performed survival analysis using two different cut-offs for each biomarker: an optimal cut-off determined by the ROC curve analysis, and a predefined cut-off recommended by the literature or the assay’s manufacturer. PENK levels and the values of all four eGFR equations differed significantly between survivors and non-survivors, whereas NGAL did not, irrespective of the cut-off applied. Of note, PENK demonstrated the strongest association with 30-day mortality, yielding the highest hazard ratio (HR) when the literature-designated cut-off of 80 pmol/L was applied (HR = 7.9; *p* < 0.001). By comparison, the four eGFR equations, when using the conventional cut-off of 60 mL/min/1.73 m^2^, yielded HRs ranging from 3.2 to 4.8 (*p* < 0.001 except for the cystatin C-based CKD-EPI equation, where *p* = 0.006) [45].

The prognostic value of PENK for 30-day mortality was further confirmed by the same research group in their prospective study which included a different cohort of 215 patients with sepsis admitted to the ER or the ICU [46]. By applying a PENK cut-off of 80 pmol/L, the authors noted that patients with elevated PENK levels had significantly poorer survival than those with PENK concentrations below the cut-off. Moreover, when dividing PENK levels into quartiles, 30-day mortality rate increased progressively across PENK quartiles, both in the overall cohort, as well as in each subgroup of patients with sepsis and septic shock. In contrast, although SOFA renal subscore was significantly associated with 30-day mortality in the total cohort, it failed to discriminate mortality risk within the sepsis and septic shock subgroups. Accordingly, the authors concluded that PENK provided more consistent prognostic stratification than the SOFA renal subscore, by accurately identifying high-risk patients even within the same stage of sepsis or septic shock. In other words, within the sepsis group, elevated PENK levels could accurately identify patients who were at increased risk of death, and inversely, within the septic shock group, lower PENK levels could consistently identify those patients who had a comparatively more favorable prognosis [46].

In a subgroup of ICU patients enrolled in the ALBIOS study, Caironi et al. evaluated the prognostic performance of PENK in terms of predicting 90-day mortality, by utilizing serial measurements of PENK [41]. During a 3-month follow-up, 369 out of 956 septic patients (38.6%) died. Kaplan–Meier analysis demonstrated a stepwise increase in the risk of 90-day mortality as PENK concentrations were gradually increasing. After adjusting for conventional clinical predictors, including SOFA score, serum lactate concentration, serum creatinine, and central venous oxygen saturation, baseline PENK levels remained an independent predictor of 90-day mortality. Furthermore, when applying serial measurements over the time course of the first 7 days, the association between PENK levels and 90-day mortality became even more pronounced [41].

In the multinational study by Hollinger et al., 28-day mortality was one of the trial’s secondary endpoints [47]. Over the follow-up period of 28 days, 127 patients from the derivation cohort (Kid-SS) died, yielding a 28-day mortality of 22%, whereas in the validation cohort (FROG-ICU) 137 (26%) deaths were recorded. High PENK levels upon admission were associated with dismal prognosis in both cohorts. In univariate analysis, PENK demonstrated better discrimination for 28-day mortality than serum creatinine, eGFR, and age, with a C-index of 0.661 compared with 0.589, 0.589, and 0.603, respectively. Furthermore, baseline PENK concentrations remained independently associated with 28-day mortality after adjustment for age, sex, comorbidities, serum lactate, serum creatinine, and sepsis severity. PENK also provided prognostic information independent of SOFA and APACHE II scores, suggesting that it may offer complementary prognostic information for mortality prediction beyond established measures of renal function and clinical disease severity [47].

Similar results regarding 28-day mortality were reported by Rosenqvist et al. [48]. Their study included 588 patients presenting to the ED with sepsis, of whom 50 patients (8.5%) died within 28 days of admission. The number of deaths increased progressively across PENK concentration quartiles. Higher PENK concentrations were independently associated with 28-day all-cause mortality, even after adjusting for eGFR. Notably, PENK outperformed serum creatinine and eGFR in predicting mortality, while also providing prognostic information beyond renal function [48].

Short-term mortality was the primary endpoint of the study by Verras et al., which included 61 patients presenting to the ED with septic shock who were followed throughout their hospitalization [50]. PENK levels were measured upon admission using a POC device. During follow-up, 39 patients (64%) died. PENK levels on admission were significantly higher in non-survivors compared to survivors. ROC analysis demonstrated a fair predictive performance of POC-PENK for in-hospital mortality (AUC = 0.723; *p* = 0.004). A cut-off value of 83.1 pmol/L identified non-survivors with 95% sensitivity and 32% specificity. Furthermore, in multivariable logistic regression analysis, logPENK remained an independent predictor of in-hospital mortality, even after adjustment for clinical and laboratory variables. When comorbidities were included, both diabetes mellitus and logPENK remained independently associated with mortality, with PENK demonstrating the strongest predictive effect [50].

A prospective observational study, which included 177 patients presenting to the ED with sepsis, evaluated the prognostic value of POC Bio-adrenomedullin (Bio-ADM) and PENK for predicting 30-day mortality [53]. At 1-month follow-up, 24.3% of patients had died. Admission PENK levels were significantly higher in non-survivors than in survivors (median 135 vs. 99 pmol/L; *p* < 0.05). Patients with PENK levels above the predefined cut-off had a significantly higher 30-day mortality. However, PENK demonstrated only modest predictive performance for 30-day mortality (AUC 0.60) and was outperformed by Bio-ADM (AUC 0.73) and the Rapid Emergency Medicine Score (REMS) (AUC 0.73) [53].

Finally, following a rather unusual case–control study design, Ibrahim et al. investigated the prognostic role of PENK in 50 ICU patients with sepsis-associated AKI [54]. The authors reported that PENK levels were significantly higher in non-survivors compared to survivors, as well as in those patients who failed to recover renal function. In multivariable Cox regression analysis, PENK and SOFA score emerged as independent predictors of overall survival, whereas eGFR was not independently associated with mortality [54].

In contrast, PENK was not identified as a predictor of mortality in the study by Ampulembang et al. [49]. In this study, serial PENK measurements were performed on admission (day 0) and on day 7 in 40 septic patients followed throughout hospitalization. Among survivors, PENK levels showed a slight decrease from admission to day 7 (454.12 vs. 416.70 pg/mL); however, this difference was not statistically significant (*p* = 0.569). Conversely, in patients who died, PENK levels increased from admission to day 7 (227.12 vs. 297.89 pg/mL), yet this change was likewise not statistically significant (*p* = 0.209). These findings indicate that temporal changes in PENK concentrations during the first week of hospitalization were not significantly associated with mortality in septic patients [49].

Frigyesi et al. also explored the prognostic role of PENK for predicting 30-day mortality [51]. Their multicenter retrospective study involved a mixed ICU population of 1978 ICU patients, 632 of whom were classified as having sepsis. Higher PENK concentrations were consistently associated with increased mortality risk in the overall ICU cohort and across clinical subgroups, including sepsis, cardiac arrest, and trauma patients. In the entire ICU population, PENK predicted 30-day mortality with moderate accuracy (AUC 0.71). Its predictive performance varied among subgroups, showing limited discriminatory ability in sepsis (AUC 0.61), moderate performance in cardiac arrest (AUC 0.73), and stronger predictive ability in trauma patients (AUC 0.84). After adjustment for SAPS-3, higher PENK levels remained independently associated with mortality in the overall population, cardiac arrest, and trauma subgroups, but not in patients with sepsis. Adding PENK to SAPS-3 did not significantly improve mortality prediction in the overall ICU population or sepsis and trauma subgroups, although a modest improvement was observed in cardiac arrest patients [51].

Table 1 summarizes the main characteristics and principal findings of studies investigating the role of PENK as a biomarker of renal dysfunction, risk stratification, and mortality in patients with sepsis.

## 5. Discussion

Despite advances in biomedical research and clinical care, sepsis-related morbidity and mortality remain high, posing a major global burden on human health and healthcare systems. Early recognition and timely initiation of appropriate management are therefore essential and should begin at the earliest point of clinical assessment, including upon presentation to the ED or admission to the ICU [1,4,5,7,15,17]. Accordingly, a substantial body of scientific research has focused on identifying reliable biomarkers and clinical scoring systems to facilitate early diagnosis, accurate risk stratification and appropriate therapeutic decision-making in patients with sepsis [18,19,20,21,22,23,24,25].

PENK has been primarily investigated as a biomarker of sepsis-associated renal dysfunction. Several studies have evaluated its ability to predict AKI development and the subsequent need for RRT in both ICU [41,42,46,47,51,52,54] and ED [44,46,48,49,50,52,53] settings. Specifically, PENK levels on admission have been shown to predict the occurrence of sepsis-associated AKI [41,44,45,47,48,49,52,54], the future need for RRT [41,43,45,46,47,51,54], major adverse kidney events [47], the occurrence of worsening renal function and/or renal failure [44,47,48,53,54], and the development of multiple organ failure [48]. In parallel, it has been reported that PENK can predict the recovery of renal function [41], the severity of sepsis [44,46] and the presence of neurological dysfunction [51]. PENK has demonstrated significant correlations with established markers of renal function, including serum creatinine [41,42,50,53,54], creatinine clearance [44], eGFR [42,43,45,46], and SOFA renal subscore [41,46,47,48]. In addition, PENK has been found to be associated with several biomarkers, namely procalcitonin [50], CRP [54], and lactate [50,53]. Notably, compared to NGAL, another novel biomarker of renal injury, PENK has demonstrated comparable or, in some settings, superior predictive performance for renal dysfunction in sepsis [44,45]. Moreover, PENK appeared to be less influenced by the systemic inflammatory response than NGAL, suggesting that the two biomarkers may provide complementary information regarding renal dysfunction [44,45].

The observed associations of PENK with serum creatinine, creatinine clearance, and eGFR, together with its inverse relationship with directly measured GFR, support its potential to reflect glomerular filtration and renal function [42,43,44,45,46]. Given that PENK may detect evolving renal dysfunction before it is fully captured by conventional markers, its measurement could potentially enable earlier identification of patients at risk of AKI and its associated complications. These findings are further supported by the favorable biological characteristics of PENK as a renal biomarker, including its relatively long half-life, its stability after sample collection, and its predominant dependence on renal filtration rather than demographic characteristics or systemic inflammation [27,31]. Moreover, its exclusive renal filtration through the glomerulus provides a physiological basis for its use as a marker of GFR, including under non-steady-state conditions encountered in critically ill patients [43,46]. Importantly, the availability of rapid POC measurement may further enhance the clinical utility of PENK, particularly in the ED, where early identification of patients at risk of renal deterioration may facilitate closer monitoring and timely intervention.

Nevertheless, the potential added value of PENK beyond established markers of renal dysfunction should be interpreted with caution. Although several studies have demonstrated that PENK provides incremental information beyond serum creatinine, eGFR, NGAL, or established clinical parameters, this finding has not been consistently reproduced across all cohorts. For instance, PENK provided incremental predictive information beyond NGAL for kidney dysfunction in one study [44] and demonstrated additional value beyond serum creatinine for predicting AKI and major adverse kidney events in selected populations [41,47]. However, in other studies, its discriminatory performance was comparable or inferior to that of established measures of renal function. In particular, the association between PENK and AKI was attenuated after adjustment for eGFR in one prospective cohort, with PENK showing inferior discrimination compared with serum creatinine and eGFR [48]. Similarly, PENK did not significantly improve the prediction of renal failure or RRT when added to creatinine in the study by Frigyesi et al. [51]. Moreover, although PENK demonstrated higher predictive performance than lactate for AKI in one study [52], the addition of lactate to PENK did not further improve discrimination, highlighting that the incremental value of combining PENK with established biomarkers may depend on the clinical setting and outcome being assessed. Taken together, these findings indicate that PENK should not be considered a universally superior biomarker of renal dysfunction, but rather a potentially complementary marker whose added clinical value appears to vary according to the population, comparator, and outcome examined. Its potential clinical utility may therefore lie primarily in combination with established clinical and laboratory parameters, particularly within a multi-marker or integrated risk-stratification approach.

Apart from its role in assessing renal dysfunction, the prognostic value of PENK in terms of mortality has also been extensively investigated. Several studies have demonstrated an association between elevated PENK concentrations and early in-hospital [44,50], mid-term [45,46,47,48,53], and long-term mortality [41]. Nevertheless, these findings have not been consistent across all studies, with some reporting limited predictive performance or no significant association between PENK and mortality [49,51]. As a prognostic marker of mortality, PENK has been evaluated in comparison with established clinical severity scores, including APACHE II [44,47], SOFA [41,46,47,50,53,54], SAPS-3 [51], and REMS [53]. Overall, the evidence regarding the prognostic value of PENK beyond established severity scores remains heterogeneous. Although PENK provided prognostic information independent of APACHE II and SOFA in some studies [41,47], other studies demonstrated an association between PENK and mortality without establishing clear incremental value beyond these scores [44,50,54]. Moreover, PENK showed more consistent prognostic stratification than the SOFA renal subscore in one study [46], but was outperformed by SOFA and REMS in another [53]. In the study by Frigyesi et al., PENK demonstrated limited discriminatory ability for mortality in the sepsis subgroup and did not improve prediction beyond SAPS-3 [51]. Importantly, the substantially higher discriminatory performance observed in trauma and cardiac arrest patients in the same study raises the possibility that the prognostic value of PENK is not specific to sepsis. Instead, PENK may reflect a broader pathophysiological response to critical illness, which can occur across diverse clinical conditions. Thus, while PENK may provide useful prognostic information in septic patients, its prognostic role should be interpreted as a marker of renal and systemic severity rather than as a sepsis-specific biomarker.

Several methodological and clinical limitations should be taken into account when interpreting the evidence regarding the clinical utility of PENK in sepsis and septic shock. The reviewed studies showed considerable heterogeneity in terms of sample size, ranging from small [42,49,50,54] to moderate [45,46,52,53] and large cohorts [41,47,48,51]. Furthermore, there were variations in study settings, including single-center [42,43,44,45,46,48,49,50,52,53,54], multicenter [41,47,51], or even multinational studies [47]. Moreover, differences in the study design, including prospective [41,42,43,46,47,48,49,50,53] and retrospective approaches [44,45,51,52], may have contributed to variability in the reported results. The observational nature of most of the studies is a key limitation, because it precludes causal inference and restricts interpretation of the observed associations between PENK levels and subsequent clinical outcomes. The studies also differed in their enrollment criteria. Ten of the fourteen studies enrolled only patients with sepsis and/or septic shock [41,42,44,45,46,47,48,49,52,53], whereas one study included exclusively patients with septic shock [50], and another included a predominantly septic shock population [43]. One case–control study evaluated patients with sepsis alongside healthy controls [54], while another study investigated a mixed cohort of critically ill patients [51]. The latter study [51] evaluated not only patients with sepsis, but also patients with cardiac arrest and trauma, along with a remaining total of 999 patients who did not present with any of these conditions. Moreover, an additional confounding factor related to recruitment was the variability in the diagnostic criteria used to define sepsis and septic shock. Specifically, eight studies used the Sepsis-III criteria [43,46,49,50,51,52,53,54], while five used former definitions [41,42,44,47,48]. One retrospective study [45] used mixed definitions, as patients were initially enrolled according to the 2012 Surviving Sepsis Campaign (SSC) criteria and were subsequently reclassified according to the Sepsis-III criteria. Regarding the clinical setting, six studies evaluated PENK exclusively in ICU patients [41,42,43,47,51,54], whereas five studies included only patients presenting to the ED [44,48,49,50,53]. Two studies were conducted in both the ED and ICU settings [46,52], while one did not specify the clinical setting of patient recruitment [45]. Furthermore, there were variations in the definition of primary and secondary endpoints. In addition, variations in the timing of patient enrollment and in the biological sample collection schedules among studies may also have introduced selection and measurement bias, as PENK levels can be influenced by the stage of disease progression at the time of assessment. Most studies examining the clinical utility of PENK relied on a single admission measurement [42,43,45,46,48,50,52,53,54], whereas a limited number of studies incorporated serial PENK measurements [41,44,47,49]. Another limitation related to sample collection concerns the timing of the admission measurement. In most studies, PENK assessment was performed within the first 24 h of admission, generally before the initiation of therapeutic interventions and/or medications. However, this relatively broad time window may introduce considerable variability in the timing of biomarker measurement relative to disease onset and progression. All but two studies, which did not report the blood analysis technique used [41,42], applied the same manufacturer’s assay for PENK measurement. Additionally, two studies utilized POC devices for PENK assessment [50,53]. However, the studies differed in the level of methodological detail provided, particularly regarding analytical performance characteristics such as turnaround time, assay sensitivity, and lower limit of detection.

Overall, the available evidence suggests that PENK may be a useful biomarker for the early assessment of renal dysfunction and prediction of sepsis-associated AKI, but its clinical value beyond established markers such as serum creatinine, eGFR, NGAL, and clinical severity scores remains incompletely established. Although several studies demonstrated incremental prognostic information, particularly for AKI, RRT requirement, and adverse renal outcomes, others reported only modest discriminatory performance or limited additional value when PENK was combined with conventional markers. Similarly, although elevated PENK concentrations have been associated with mortality in both ICU and ED populations, its prognostic performance has been inconsistent and generally moderate, with some studies failing to demonstrate significant associations or meaningful improvement beyond established severity scores. Of note, the observation that PENK showed greater discriminatory performance for mortality in trauma and cardiac arrest than in sepsis could support the notion that PENK reflects renal dysfunction and overall disease severity through pathophysiological pathways of organ dysfunction and critical illness that are not unique to sepsis. Thus, PENK may be best regarded as a complementary biomarker that could provide additional information in selected patients, rather than as a standalone or sepsis-specific marker. The availability of rapid POC assays may facilitate its incorporation into early clinical assessment; however, further prospective studies are needed to determine whether PENK-guided assessment improves risk stratification or clinical decision-making beyond currently available biomarkers and severity scores.

Furthermore, the increasing recognition of sepsis heterogeneity has strengthened the rationale for a precision-medicine approach. The development and validation of patient-specific biomarkers may allow for more individualized diagnostic and prognostic approaches by enabling the characterization of distinct clinical and biological phenotypes within sepsis [60]. However, the incorporation of novel biomarkers into routine clinical practice remains challenging. Factors such as cost, availability, variability in assay performance, and the absence of standardized measurement procedures may limit the widespread adoption of such biomarkers. Furthermore, although rapid POC testing could facilitate timely biomarker assessment in the ED and ICU, these technologies require further analytical and clinical validation before their routine use can be recommended.

## 6. Conclusions

Sepsis remains associated with substantial morbidity and mortality, underscoring the need for combined clinical assessment, scoring systems and biomarkers that would support early diagnosis, risk stratification and management. Current evidence suggests that PENK may provide useful information regarding renal dysfunction, sepsis-associated AKI, and mortality; however, its prognostic and diagnostic performance in the ED and ICU settings has been variable, and its incremental value beyond established biomarkers and clinical severity scores remains uncertain. PENK may therefore have potential as a complementary biomarker, particularly when interpreted alongside established clinical and laboratory parameters, rather than as a standalone marker. Further large, well-designed prospective studies are needed to clarify its added clinical value, determine which patient populations may benefit most from its measurement, and establish whether its incorporation into clinical decision-making improves patient outcomes.

## Figures and Tables

**Table 1:** Overview of clinical studies evaluating PENK in sepsis and its association with renal and prognostic outcomes.

Author (Year), Country	Study Design and Clinical Setting	Study Population	Main Findings
Doemming et al. (2015), Germany [42]	Prospective observational single-center pilot study, ICU	• Total cohort: 42 surgical pts with sepsis; 67% male • 3 subgroups: Sepsis: 8 pts (19%); Severe sepsis: 19 pts (45%); Septic shock: 15 pts (36%)	• PENK levels did not differ significantly among sepsis severity subgroups. • Strong correlation of PENK with eGFR, moderate correlation with sCr. • Higher PENK levels in pts with renal dysfunction and metabolic acidosis.
Beunders et al. (2020), Netherlands [43]	Prospective observational, single-center, pilot study, ICU	• Overall cohort: 23 septic pts; 65% male • Septic shock: 20 pts (87%)	• eGFR_MDRD_ and GFR_ECC_ were both correlated with the GFR_iohexol_, which was regarded as the reference (“true”) GFR, but both tended to overestimate renal function, resulting in inaccurate GFR classification. • PENK concentrations were strongly correlated with the GFR_iohexol_, demonstrating greater accuracy than eGFR_MDRD_ and GFR_ECC_.
Marino et al. (2015), Italy [44]	Retrospective observational single-center study, ED	• Overall cohort: 101 pts with suspected sepsis; 60% male • Severe sepsis or septic shock: 29 pts (29%)	• PENK was more specific than NGAL for assessing AKI in septic patients. •PENK levels correlated with sepsis severity and creatinine clearance, and increased progressively with advancing RIFLE stages of AKI severity • NGAL had comparable diagnostic accuracy for AKI, but its levels were markedly affected by inflammation. • PENK and NGAL demonstrated fair prognostic performance for 7-day mortality, outperforming creatinine clearance but showing lower prognostic accuracy than APACHE II score. Serial PENK measurements further improved prognostic performance.
Kim et al. (2017), Korea [45]	Cross-sectional retrospective observational single-center registry study, Clinical setting not defined	• Overall cohort: 167 septic pts; 59.3% male [Enrollment period: December 2014–June 2015] • Suspected sepsis: 31 pts • Sepsis: 99 pts • Septic shock: 37 pts	• PENK, NGAL, and all four eGFR values were significantly associated with sepsis severity and differed significantly between pts with and without AKI only in the sepsis subgroup. • Unlike NGAL, PENK levels were not influenced by systemic inflammation. • PENK outperformed NGAL in predicting AKI and RRT. • PENK was superior to NGAL in predicting 30-day mortality. • PENK showed consistent and significant associations with all four eGFR equations, irrespective of the eGFR equation used.
Kim et al. (2020), Korea [46]	Prospective observational single-center registry study, ICU and ED	• Total cohort: 215 septic pts; 59.1% male[Enrollment period: August 2016–August 2017] • Sepsis: 109 pts (50.7%) • Septic shock: 106 pts (49.3%)	• Higher PENK levels on admission were significantly associated with septic shock, use of vasopressors, need for RRT on day 1, and 30-day mortality. • PENK levels increased progressively with worsening SOFA renal subscore and declining eGFR. • PENK could predict the need for RRT better than the SOFA renal subscore. • PENK quartiles were associated not only with progressively worse renal function, but also with an increasing trend in the number of organ failures. • PENK quartiles demonstrated superior prognostic performance, compared to the SOFA renal subscore, in terms of predicting and stratifying 30-day mortality in each subgroup of patients with sepsis and septic shock.
Caironi et al. (2018), Italy [41]	Prospective observational multi-center study (ALBIOS study), ICU	• Overall cohort: 956 septic pts • Septic shock: 539 pts (56.4%)	• PENK levels on admission correlated with sCr and increased progressively with worsening SOFA renal subscore. • PENK was independently associated with markers of renal dysfunction and overall disease severity, including higher sCr, lower urinary output, higher total SOFA and SAPS II scores, CKD, mechanical ventilation, lower FiO2, and lower MAP. • PENK independently predicted both early (within 48 h) and mid-term (within 7 days) incident AKI. • Serial PENK measurements further improved prediction of AKI. • PENK independently predicted both the immediate and subsequent need for RRT and provided incremental predictive value beyond serum creatinine. • Lower baseline PENK levels were associated with short-term renal recovery, accurately predicting improvement in renal function within both 48 h and 7 days. • Baseline PENK independently predicted 90-day mortality after adjustment for SOFA, Lac, sCr, and central venous oxygen saturation. Serial PENK measurements during the first week further improved prognostic accuracy for 90-day mortality.
Hollinger et al. (2018), Multinational (France, Belgium, Netherlands, Italy, Germany) [47]	Prospective observational multicenter study (“Kid-SS”—sub-study of ADRENOSS-I), ICU	• Sepsis cohort of Kid-SSS Study: 583 pts; 62% male - Septic shock: 293 pts (50.3%) • Validation cohort of FROG-ICU study: 525 septic pts; 66.1% male - Septic shock: 478 pts (91%)	• PENK levels on admission independently predicted the occurrence of MAKE, AKI and WRF within 7 days. • Baseline PENK levels increased progressively with increasing AKI severity and were independently associated with the need for RRT. • PENK provided incremental predictive value beyond sCr and demonstrated superior performance compared to creatinine in pts with low baseline sCr or without CKD. • Serial PENK measurements further improved prediction of MAKE and WRF. • Low baseline PENK levels could identify pts with AKI who were more likely to recover, outperforming sCr in predicting transient AKI and early renal recovery. • Baseline PENK concentrations independently predicted 28-day mortality in both the derivation and validation cohorts and showed better prognostic performance than sCr and eGFR.
Rosenqvist et al. (2019), Sweden [48]	Prospective observational, single-center study, ED	• Overall cohort: 588 septic pts; 51% male • Septic shock: 21 pts (3.7%)	• Baseline PENK was associated with AKI development at 48 h and 7 days, although this association was attenuated after accounting for baseline eGFR. • Among pts with normal to mildly impaired renal function, PENK levels were predictive of worsening renal function, even after adjusting for eGFR. • Admission PENK levels independently predicted the development of MOF, providing prognostic information beyond eGFR. • PENK independently predicted 28-day mortality and outperformed sCr and eGFR for mortality prediction.
Ampulembang et al. (2024), Indonesia [49]	Prospective observational single-center study, ED	• Overall cohort: 40 septic pts without AKI on admission; 57.5% male • SI-AKI within 7 days: 16 pts (40%) • no AKI within 7 days: 24 pts (60%)	• Baseline PENK levels showed a weak, non-significant association with AKI incidence. • PENK levels on admission significantly discriminated AKI development in septic pts, albeit with moderate predictive performance. • PENK levels increased significantly from admission to day 7 in pts who developed AKI, whereas they decreased significantly over the same period in those without AKI. • Changes in PENK levels over the first 7 days were not significantly associated with survival outcome in septic pts.
Verras et al. (2024), Greece [50]	Prospective observational single-center study, ED	• Overall cohort: 61 pts with septic shock; 47% male	• PENK correlated positively with creatinine, procalcitonin, lactate, SuPAR, and SOFA score. • Baseline PENK levels were not significantly different between pts with and without AKI and did not predict AKI development. • PENK levels on admission were significantly higher in non-survivors than survivors and predicted in-hospital mortality. • Log-transformed PENK remained an independent predictor of in-hospital mortality after adjustment for clinical and laboratory variables.
Frigyesi et al. (2021), Sweden [51]	Retrospective observational, multicenter study, ICU	• Total cohort: 1978 pts; 61% male • Sepsis subgroup: 632 pts (32%) • Cardiac arrest subgroup: 190 pts (9.6%) • Trauma subgroup: 157 pts (7.9%)	• Higher admission PENK levels were associated with greater day-2 organ dysfunction and higher SOFA scores. • PENK correlated with renal, cardiovascular, hepatic, and neurological SOFA subscores. • After adjustment for sCr, PENK remained associated with cardiovascular and neurological dysfunction, particularly in septic pts. • PENK predicted day-2 renal dysfunction, but sCr showed better discrimination. • PENK was associated with RRT requirement; however, adding PENK to sCr did not improve predictive accuracy. • Higher admission PENK levels were independently associated with increased 30-day mortality. • PENK predicted 30-day mortality with moderate accuracy (AUC 0.71). Predictive performance varied by subgroup: sepsis (AUC 0.61), cardiac arrest (AUC 0.73), and trauma (AUC 0.84). • After adjustment for SAPS-3, PENK remained an independent predictor of mortality in the overall ICU population, cardiac arrest, and trauma pts, but not in sepsis. • Adding PENK to SAPS-3 improved mortality prediction only in cardiac arrest pts; no significant improvement was observed in the overall ICU, sepsis, or trauma subgroups.
Ji et al. (2025), China [52]	Retrospective observational, single-center study, ED and ICU	• Overall cohort: 161 septic pts; 71.4% male • AKI group: 66 pts (41%) • No-AKI group: 95 pts (59%)	• Patients with AKI had significantly higher PENK levels than those without AKI. • PENK and Lac levels on admission were independent predictors of AKI development. • PENK showed superior predictive performance compared to Lac.
Casalboni et al. (2022), Italy [53]	Prospective observational single-center study, ED	• Overall cohort: 177 septic pts; 44.6% male	• Higher admission PENK levels were associated with impaired renal function, including reduced urine output and higher sCr. • Elevated PENK was also associated with higher SOFA scores, higher Lac, BNP, and potassium levels, and lower blood pH. • Admission PENK levels were significantly higher in non-survivors than survivors. • PENK showed modest discrimination for 30-day mortality and was outperformed by bio-ADM and REMS.
Ibrahim et al. (2022), Egypt [54]	Case–control single-center study, ICU	• Overall cohort: 80 subjects; 43.75% male • SI-AKI group: 50 ICU pts (62.5%) • Control (no AKI) group: 30 healthy subjects (37.5%)	• PENK levels on admission were significantly higher in the SI-AKI group compared to healthy controls. • PENK was correlated with sCr, CRP, SIRS and SOFA scores. • Higher admission PENK levels predicted the need for RRT. • PENK could better predict non-recovery of AKI (CKD and/or death) than sCr, eGFR, and CRP. • PENK was an independent predictor of overall survival, together with the SOFA score, whereas eGFR was not independently associated with mortality.

Abbreviations: AKI = Acute kidney injury; ALBIOS = Albumin Italian outcome sepsis study; APACHE II = Acute physiology and chronic health evaluation II; AUC = Area under the curve; bio-ADM = Bio-adrenomedullin; BNP = B-type natriuretic peptide; CI CKD = Chronic kidney disease; CRP = C-reactive protein; ECC = Endogenous creatinine clearance; ED = Emergency department; eGFR = Estimated glomerular filtration rate; FROG-ICU = French and European out-come registry in intensive care unit; ICU = Intensive care unit; Kid-SSS = Kidney in sepsis and septic shock study; Lac = Lactate; MAP = Mean arterial pressure; MAKE = Major adverse kidney event; MDRD = Modification of diet in renal disease equation; MOF = Multiple organ failure; NGAL = Neutrophil gelatinase–associated lipocalin; NPV = Negative predictive value; PENK = Proenkephalin; pts = patients; REMS = Rapid emergency medicine score; RIFLE = Risk, injury, failure, loss of function, end-stage renal disease; RRT = Renal replacement therapy; SAPS II= Simplified acute physiology score II; sCr = Serum creatinine; SI-AKI = Sepsis-induced acute kidney injury; SIRS = Systemic inflammatory response syndrome; SOFA = Sequential organ failure assessment; SuPAR = Soluble urokinase plasminogen activator receptor; WRF = Worsening renal function.

## Data Availability

No new data were created or analyzed in this study. Data sharing is not applicable to this article.

## Abbreviations

The following abbreviations are used in this manuscript:

ADH	Antidiuretic Hormone
ADQI	Acute Disease Quality Initiative
AdrenOSS	Adrenomedullin and Outcome in Severe Sepsis and Septic Shock trial
AKI	Acute Kidney Injury
ALBIOS	Albumin Italian Outcome Sepsis study
AUC	Area Under the Curve
bio-ADM	Bio-Adrenomedullin
BMI	Body Mass Index
BNP	B-type Natriuretic Peptide
CKD-EPI	Chronic Kidney Disease Epidemiology Collaboration
CRP	C-Reactive Protein
ED	Emergency Department
eGFR	Estimated Glomerular Filtration Rate
FROG-ICU	French and European Outcome Registry in Intensive Care Unit
GFR	Glomerular Filtration Rate
HR	Hazard Ratio
ICU	Intensive Care Unit
IQR	Interquartile Range
Kid-SSS	Kidney in Sepsis and Septic Shock study
MDRD	Modification of Diet in Renal Disease study
NGAL	Neutrophil Gelatinase-Associated Lipocalin
PENK	Proenkephalin
POC	Point-of-Care
pPENK-A	Preproenkephalin A
REMS	Rapid Emergency Medicine Score
RIFLE	Risk, Injury, Failure, Loss of function, End-stage renal disease
ROC	Receiver Operating Characteristic
RRT	Renal Replacement Therapy
SAPS II	Simplified Acute Physiology Score II
SIRS	Systemic Inflammatory Response Syndrome
SOFA	Sequential Organ Failure Assessment
SSC	Surviving Sepsis Campaign
SuPAR	Soluble Urokinase Plasminogen Activator Receptor
WHO	World Health Organization
